# Down-regulation of TGF-**β** receptors in human colorectal cancer: implications for cancer development

**DOI:** 10.1038/sj.bjc.6690339

**Published:** 1999-04

**Authors:** M Matsushita, K Matsuzaki, M Date, T Watanabe, K Shibano, T Nakagawa, S Yanagitani, Y Amoh, H Takemoto, N Ogata, C Yamamoto, Y Kubota, T Seki, H Inokuchi, M Nishizawa, H Takada, T Sawamura, A Okamura

**Affiliations:** Third Department of Internal Medicine, Kansai Medical University, Moriguchi, Osaka, Japan; Department of Ophthalmology, Kansai Medical University, Moriguchi, Osaka, Japan; Department of Gastroenterology, Saiseikai Noe Hospital, Osaka, Japan; Department of Medical Chemistry, Kansai Medical University, Moriguchi, Osaka, Japan; Second Department of Surgery, Kansai Medical University, Moriguchi, Osaka, Japan; Department of Surgical Pathology, Kansai Medical University, Moriguchi, Osaka, Japan

**Keywords:** TGF-β, TGF-β, receptor, Smad, colorectal cancer, colorectal adenoma

## Abstract

Many colorectal cancer cells are resistant to the anti-proliferative effects of transforming growth factor-β (TGF-β). TGF-β also acts as paracrine factor from cancer cells on their mesenchymal cells. The aim of this study was to examine the expression of TGF-β and its receptors in human colorectal cancer tissue and determine any relationship with cancer growth. In situ hybridization and Northern blot hybridization detection of TGF-β_1_, type I and type II receptor mRNA and immunohistochemical staining of TGF-β_1_ were performed using 11 human colorectal adenomas, 22 colorectal cancers and ten normal colorectal mucosas as control. TGF-β receptor mRNAs were expressed mainly by normal colorectal epithelial cells and adenoma. However, mRNAs for TGF-β receptors were only faintly, if at all, expressed in eight of 22 human colorectal cancers. In addition, intense signals of TGF-β_1_ mRNA and the protein were detected in all colorectal cancers. TGF-β receptor mRNAs and TGF-β_1_ protein were also distributed in fibroblasts and endothelial cells in the interstitium. Moreover, Smad 4 protein was translocated to nucleus in primarily cultured adenoma cells, but not in cancer cells after TGF-β stimulation. The escape of human colon cancer from TGF-β -mediated growth inhibition by down-regulation of TGF-β receptors as well as the effects of TGF-β on stroma formation and angiogenesis indicate a possible role for TGF-β in the progression of colon cancer in an intact host. © 1999 Cancer Research Campaign


*British Journal of Cancer* (1999) **80**(1/2), 194–205
© 1999 Cancer Research Campaign
Article no. bjoc.1998.0339

### Down-regulation of TGF-β receptors in human colorectal cancer: implications for cancer development

**M Matsushita[1], K Matsuzaki[1], M Date[1], T Watanabe[1], K Shibano[1], T Nakagawa[1], S Yanagitani[1], Y Amoh[1], H Takemoto[1], N Ogata[2], C Yamamoto[2], Y Kubota[1], T Seki[1], H Inokuchi[3], M Nishizawa[4], H Takada[5], T Sawamura[1], A Okamura[6] and K Inoue[1]**

[1]Third Department of Internal Medicine, Kansai Medical University, Moriguchi, Osaka, Japan; [2]Department of Ophthalmology, Kansai Medical University, Moriguchi, Osaka, Japan; [3]Department of Gastroenterology, Saiseikai Noe Hospital, Osaka, Japan; [4]Department of Medical Chemistry, Kansai Medical University, Moriguchi, Osaka, Japan; [5]Second Department of Surgery, Kansai Medical University, Moriguchi, Osaka, Japan; [6]Department of Surgical Pathology, Kansai Medical University, Moriguchi, Osaka, Japan

**Summary** Many colorectal cancer cells are resistant to the anti-proliferative effects of transforming growth factor-β (TGF-β). TGF-β also acts as paracrine factor from cancer cells on their mesenchymal cells. The aim of this study was to examine the expression of TGF-β and its receptors in human colorectal cancer tissue and determine any relationship with cancer growth. In situ hybridization and Northern blot hybridization detection of TGF-$\beta_1$, type I and type II receptor mRNA and immunohistochemical staining of TGF-$\beta_1$ were performed using 11 human colorectal adenomas, 22 colorectal cancers and ten normal colorectal mucosas as control. TGF-β receptor mRNAs were expressed mainly by normal colorectal epithelial cells and adenoma. However, mRNAs for TGF-β receptors were only faintly, if at all, expressed in eight of 22 human colorectal cancers. In addition, intense signals of TGF-$\beta_1$ mRNA and the protein were detected in all colorectal cancers. TGF-β receptor mRNAs and TGF-$\beta_1$ protein were also distributed in fibroblasts and endothelial cells in the interstitium. Moreover, Smad 4 protein was translocated to nucleus in primarily cultured adenoma cells, but not in cancer cells after TGF-β stimulation. The escape of human colon cancer from TGF-β -mediated growth inhibition by down-regulation of TGF-β receptors as well as the effects of TGF-β on stroma formation and angiogenesis indicate a possible role for TGF-β in the progression of colon cancer in an intact host.

**Keywords:** TGF-β; TGF-β receptor; Smad, colorectal cancer; colorectal adenoma

The transforming growth factor beta (TGF-β)/activin superfamily is comprised of multifunctional and ubiquitous peptides with roles in many areas of cell biology. Three different isoforms of TGF-β, designated as TGF-$\beta_1$, -$\beta_2$ and -$\beta_3$, with similar but not identical biological activities, have been identified in various mammalian tissues and cells (Barnard et al, 1990; Sporn et al, 1992). The intracellular biological effects of TGF-β are initiated following ligand binding to oligomeric complexes of high affinity TGF-β type I and type II receptors (TGF-β RI and TGF-β RII). If one of the receptors is absent or inactivated, the cells lose their responsiveness to TGF-β. cDNAs encoding mammalian type II receptor for TGF-β and activin have been cloned, and each of these receptors is a transmembrane serine/threonine kinase (Mathew et al, 1991; Lin et al, 1992). Using polymerase chain reaction (PCR) cloning approaches, a series of novel serine/threonine kinase receptors (SKRs) and activin-receptor-like kinases (ALKs) have been identified (He et al, 1993; ten Dijke et al, 1993; Matsuzaki et al, 1993; Xu et al, 1994). ALK-5 has been shown to be a signalling type I receptor for TGF-β (Franzen et al, 1993), whereas SKR1 and SKR2 have been shown to be activin type I receptors (ten Dijke et al, 1994; Xu et al, 1995; Matsuzaki et al, 1996). The type I receptor kinase domains are more similar to each other than they are to the type II receptor kinase domains. Signalling by these receptors is mediated by the recently identified Smad protein family. Upon phosphorylation by activated receptors, Smads form complexes, move into the nucleus, associate with DNA-binding proteins and activate gene transcription (Massagué et al, 1997)

One of the most prominent effects of TGF-β in vitro is a pronounced inhibition of epithelial cell growth (Robert et al, 1985). However, it is well known that cancers typically demonstrate resistance to the growth inhibitory effect of TGF-β (Serra et al, 1996). In the colorectal adenoma–carcinoma sequence (Muto et al, 1975), the conversion of the non-tumorigenic phenotype of human colonic adenoma cell lines to the tumorigenic phenotype is accompanied by a reduced response to the growth inhibitory effects of TGF-β (Manning et al, 1991). Furthermore, most colon cancer cells are resistant to the anti-proliferative effects of TGF-β (Hoosein et al, 1989). Loss of the growth inhibitory response to TGF-β at the cellular level is probably a more important step in the malignant progression. In addition, possible mechanisms by which TGF-β facilitates the progression of tumour growth include immunosuppression, angiogenesis and changes in the extracellular matrix (Sporn et al, 1988).

Modulation of growth factor effects can be achieved by various mechanisms, including changes in ligand concentration, activation of latent forms of the ligand, modulation of number and affinity of

*Received 30 December 1997*
*Accepted 26 August 1998*

*Correspondence to:* K Matsuzaki, Third Department of Internal Medicine, Kansai Medical University, Fumizonocho 10-15, Moriguchi, Osaka 570-8506, Japan

**Table 1** Clinical profiles of patients studied and mRNA expressions of TGF-$\beta_1$, TGF-β RI and TGF-β RII and TGF-$\beta_1$ immunoreactivity in human colorectal cancers

| | | | | | | mRNA expression level[b] | | | | | | Staining intensity[b] | |
|---|---|---|---|---|---|---|---|---|---|---|---|---|---|
| | | | | | | TGF-β RI | | TGF-β RII | | TGF-$\beta_1$ | | TGF-$\beta_1$ | |
| Case | Site | Tumour size (cm × cm) | pTNM[a] | pTNM stage[a] | Histology | Nor. | Ca. | Nor. | Ca. | Nor. | Ca. | Nor. | Ca. |
| 1 | Transverse | 3.2 × 4.4 | T4,N1,M0 | III | Moderate | 2+ | – | 2+ | ± | + | + | + | + |
| 2 | Sigmoid | 3.8 × 2.9 | T2,N0,M0 | I | Well | 2+ | – | 2+ | ± | + | + | + | + |
| 3 | Ascending | 4.8 × 7.4 | T3,N0,M0 | II | Well | 2+ | – | 2+ | ± | + | 2+ | + | + |
| 4 | Sigmoid | 3.8 × 4.2 | T3,N0,M0 | II | Moderate | 2+ | ± | 2+ | ± | + | + | + | 2+ |
| 5 | Rectum | 4.9 × 4.5 | T3,N1,M0 | III | Moderate | 2+ | ± | 2+ | ± | + | + | + | 2+ |
| 6 | Sigmoid | 4.0 × 4.4 | T3,N1,M1 | IV | Moderate | 2+ | ± | 2+ | ± | + | + | + | 2+ |
| 7 | Rectum | 4.8 × 7.8 | T4,N0,M0 | III | Moderate | 2+ | ± | 2+ | ± | + | + | + | 2+ |
| 8 | Sigmoid | 4.3 × 5.6 | T3,N2,M0 | III | Moderate | 2+ | ± | 2+ | ± | + | + | + | + |
| 9 | Sigmoid | 4.8 × 3.4 | T4,N1,M1 | IV | Moderate | 2+ | 2+ | 2+ | + | + | + | + | + |
| 10 | Ascending | 2.5 × 3.2 | T3,N0,M0 | II | Moderate | 2+ | 2+ | 2+ | + | + | ± | + | 2+ |
| 11 | Sigmoid | 4.5 × 6.4 | T3,N2,M0 | III | Moderate | 2+ | 2+ | 2+ | 2+ | + | + | + | 2+ |
| 12 | Rectum | 4.8 × 6.0 | T4,N2,M1 | IV | Well | 2+ | 2+ | 2+ | 2+ | + | + | + | 2+ |
| 13 | Rectum | 3.6 × 4.8 | T4,N3,M1 | IV | Moderate | 2+ | 2+ | 2+ | 2+ | + | + | + | + |
| 14 | Sigmoid | 4.0 × 5.2 | T3,N0,M0 | II | Moderate | 2+ | 2+ | 2+ | 2+ | + | + | + | + |
| 15 | Sigmoid | 4.4 × 5.4 | T3,N0,M0 | II | Well | 2+ | 2+ | 2+ | 2+ | + | + | + | 2+ |
| 16 | Ascending | 9.0 × 10.1 | T3,N0,M0 | II | Moderate | 2+ | 2+ | 2+ | 2+ | + | + | + | 2+ |
| 17 | Descending | 4.2 × 5.3 | T3,N1,M0 | III | Moderate | 2+ | 2+ | 2+ | 2+ | + | + | + | 2+ |
| 18 | Ascending | 7.3 × 4.2 | T3,N1,M0 | III | Poor | 2+ | 2+ | 2+ | 2+ | + | + | + | 2+ |
| 19 | Rectum | 4.4 × 3.7 | T3,N0,M0 | II | Moderate | 2+ | 2+ | 2+ | 2+ | + | + | + | 2+ |
| 20 | Transverse | 5.0 × 7.5 | T3,N3,M0 | III | Well | 2+ | 2+ | 2+ | 2+ | + | + | + | 2+ |
| 21 | Sigmoid | 4.3 × 5.4 | T3,N1,M0 | III | Moderate | 2+ | 2+ | 2+ | 2+ | + | + | + | 2+ |
| 22 | Descending | 4.8 × 7.2 | T3,N0,M0 | II | Moderate | 2+ | 2+ | 2+ | 2+ | + | + | + | 2+ |

Nor., normal colorectal epithelial cells; Ca., cancer cells. [a]pTNM classification. pT: extent of the primary tumour; pN: regional lymph node metastasis; pM; distant metastasis. [b]The first three parameters were graded as follows; –, no; ±, faint; +, moderate; 2 +, intense.

receptors, and alterations in post-receptor pathways. Several reports indicate that elevated levels of TGF-β mRNA and protein in colorectal cancer are associated with cancer progression (Tsushima et al, 1996). If TGF-β is indeed an inhibitor for epithelial cells, how then do colorectal cancer cells manage to proliferate despite elevated TGF-β production by tumour cells? Concerning the mechanisms of resistance to the anti-proliferative effects of TGF-β in colorectal cancer, Markowitz et al (1995) identified a specific TGF-β RII mutation that is associated with defective DNA mismatch repair in colon cancer cells. However, numerous reports also indicate that transcriptional regulation makes an important contribution to determination of the expression level of TGF-β receptors (Birchenall-Roberts et al, 1995; Kim et al, 1997). Accordingly, to clarify the mechanisms of resistance to the anti-proliferative effects of TGF-β in vivo, we estimated the transcriptional level of TGF-β receptors in colorectal cancer using in situ hybridization and Northern blot hybridization as the standard for those in normal colorectal epithelial cells.

In addition, no studies of human colorectal tumour to date have compared in situ levels of TGF-$\beta_1$ with those of its receptors as a presumptive target gene, and with the presence of ligand protein. To ascertain if TGF-β from colorectal cancer acts as paracrine factor on mesenchymal cells, we analysed the distributions of TGF-β and its receptors by a combination of in situ hybridization of TGF-$\beta_1$ receptors and its ligand, and the immunohistochemistry of TGF-$\beta_1$.

### MATERIALS AND METHODS

### Tumour specimens

Twenty-two advanced colorectal cancer specimens and 11 adenoma specimens were obtained from patients at Kansai Medical University Hospital. Ten men and 12 women with an age range of 38–81 years (mean age, 60 years at diagnosis) were included in advanced cancer cases, and seven men and four women with an age range of 45–72 years (mean age, 53 years at diagnosis) in adenoma cases. The tumours were diagnosed histologically as colorectal adenoma or cancers. Adjacent non-involved colorectal mucosa was also examined in all cases. These patients had undergone partial colorectomy or polypectomy between January 1996 and March 1998. Informed consent was obtained from every patient. In all cases, tissue samples were quickly embedded in OCT compound (Miles, Elkhart, IN, USA), frozen in dry ice–acetone and stored at –80°C until analysis.

A section from each specimen block was stained with haematoxylin–eosin for histological evaluation, and representative blocks were chosen for in situ hybridization and immunohistochemistry. In addition, non-involved colorectal mucosa adjacent to the tumour was used as an internal positive control for TGF-β and their receptors. Tumours were examined by an experienced observer without knowledge of staining results.

Eleven cases of colorectal adenoma tissues were shown in a proportion of cases with mild ($n$ = 4), moderate ($n$ = 5) and severe

© Cancer Research Campaign 1999

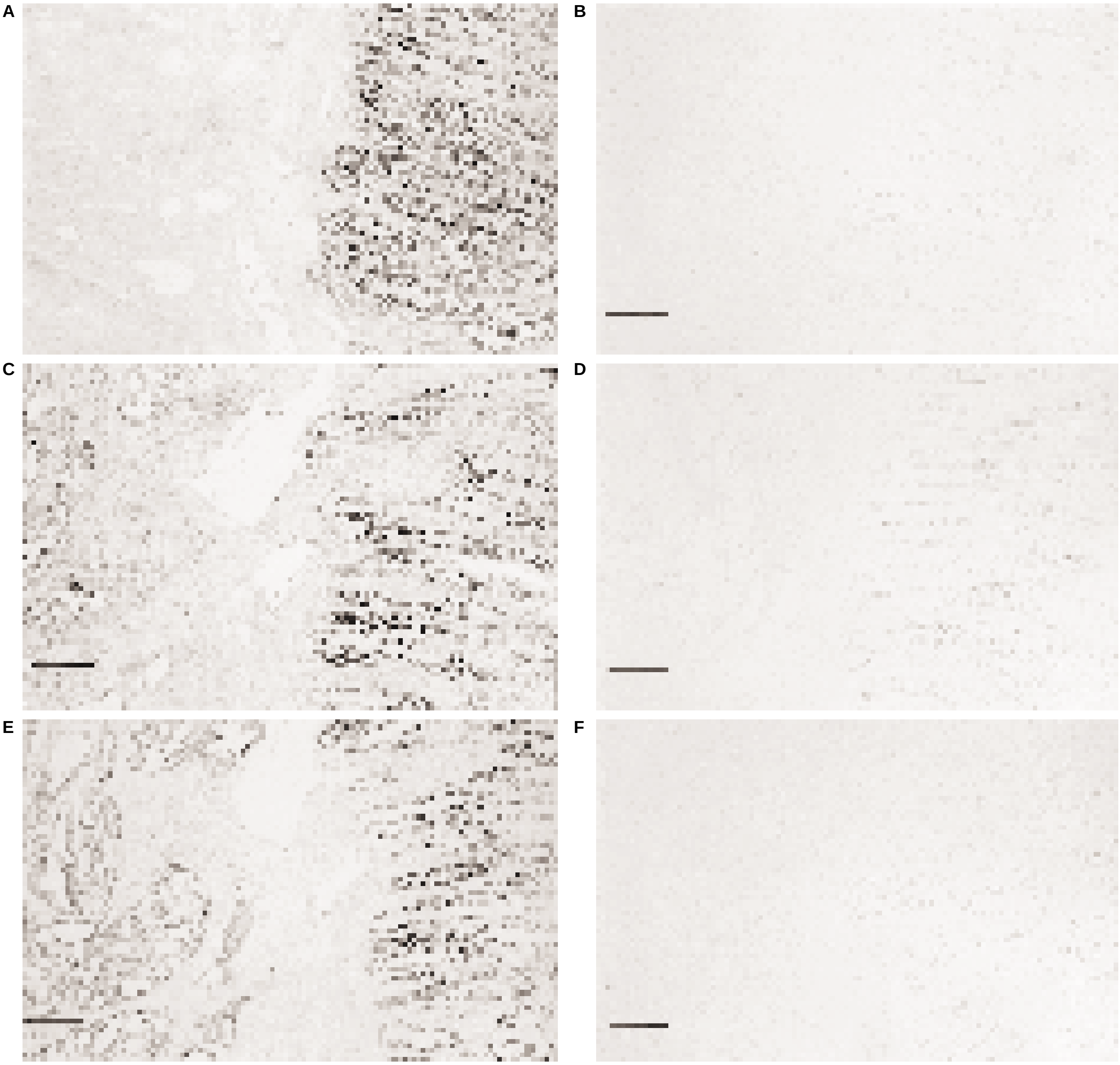

**Figure 1** Differential expression patterns of TGF-β RI, TGF-β RII and TGF-$\beta_1$ mRNA in human colon cancer and non-involved normal colon mucosa by in situ hybridization. **(A)** In situ hybridization with antisense probe to TGF-β RI shows a high level of message (mRNA expression) prominently in human normal colon epithelial cells (right side), but no mRNA expression in cancer cells (left side). **(C)** In situ hybridization with antisense probe to TGF-β RII shows high levels of mRNA prominently in normal colon epithelial cells (right side), but low levels of mRNA in cancer cells (left side). **(E)** In situ hybridization with antisense probe to TGF-$\beta_1$ shows high levels of mRNA both in human normal colon epithelial cells (right side) and in cancer cells (left side). In situ hybridization with a sense probe to TGF-β RI **(B)**, TGF-β RII **(D)** and TGF-$\beta_1$ **(F)** shows no signal above background on a sequential section of the same sample. Bars: 100 μm atypia (*n* = 2). No adenoma tissues contained cancer cells. Colorectal cancers were histologically classified as well (*n* = 5), moderately (*n* = 16) and poorly differentiated adenocarcinoma (*n* = 1). Tumours were localized at the ascending colon (*n* = 4), transverse colon (*n* = 2), descending colon (*n* = 2), sigmoid colon (*n* = 9) and rectum (*n* = 5). Duke's classification was graded as A (*n* = 9), B (*n* = 1), C (*n* = 8) and D (*n* = 4) (Table 1). The tumours were staged at the time of surgery by the standard criteria for TNM staging using the unified international colorectal cancer staging classification (Hutter et al, 1986).

### In situ hybridization

### *RNA probe*

Hybridization was performed using the following cDNA probes: 0.30-kb fragment of rat TGF-$\beta_1$ (Qian et al, 1990); 0.37-kb PCR fragment (forward primer, 5′-CGGAAGCTTACAGTGTTTCTGC-CACCTCT-3′; reverse primer, 5′-GATAAGCTTCGATGGTGAAT-GACAGTGCG-3′) of human TGF-β RI (Franzen et al, 1993); 0.61-kb PCR fragment (forward primer, 5′-TAAGGATCCTATGAC-GAGCAGCGGGGTCTG-3′; reverse primer, 5′-GGCGAATTCG-

*© Cancer Research Campaign 1999*

GTTTCCCAGGTTGAACT-3′) of human TGF-β RII (Lin et al, 1992). To avoid cross-hybridization between TGF-β RI and TGF-β RII, we chose extracellular domains of TGF-β RI and TGF-β RII cDNA. We also used the latent associated peptide portion of rat TGF-$\beta_1$ cDNA, which does not have any homology to TGF-$\beta_2$ or -$\beta_3$. All the cDNAs were subcloned into pBluescript II KS (+) vector (Stratagene Inc., La Jolla, CA, USA). Sense and antisense RNA probes were transcribed in vitro in the presence of digoxigenin-11-UTP (Boehringer Mannheim, GmbH, Mannheim, Germany). The transcription reactions were carried out for 2 h at 37°C in 20 μl of 1 × transcription buffer (50 mM Tris-HCl, 10 mM magnesium chloride ($MgCl_2$), 5 mM dithioerythritol, 0.1 mM EDTA at pH 7.2) containing 1 μg of the template DNA, 1 mM each of GTP, CTP and ATP, 0.65 mM UTP, 0.35 mM digoxigenin-11-UTP, 20 units of ribonuclease inhibitor and 20 units of T3 or T7 RNA polymerase (Boehringer Mannheim). After transcription, the DNA templates were digested with DNase I (Boehringer Mannheim) for 15 min at 37°C. Riboprobes were precipitated with ethanol and LiCl, and then resuspended in diethylpyrocarbonate-treated water.

### *Hybridization*

Frozen tissue sections (6 μm thick) were digested with 10 μg $ml^{-1}$ of proteinase K (Boehringer Mannheim) for 10 min and rinsed in phosphate-buffered saline (PBS), pH 7.4, then placed in 0.2 N HCl for 5 min at room temperature. Before hybridization, tissue sections were acetylated in a 0.25% solution of acetic anhydride in 100 mM triethanolamine, pH 8.0, for 10 min at room temperature, rinsed in PBS and dehydrated in 70, 80, 90 and 100% ethanol. Colon sections then were hybridized in 50% formamide, 10 mM Tris-HCl pH 7.6, 250 ng $\mu l^{-1}$ yeast RNA, 250 ng $\mu l^{-1}$ salmon sperm DNA, 1 × Denhardt's solution, 10% dextran sulfate, 600 mM NaCl and 1–2 μg $\mu l^{-1}$ digoxigenin-labelled riboprobe for 16 h at 50°C in a formamide-saturated humidified chamber. Subsequently, hybridization products were rinsed in 5 × saline–sodium citrate (SSC) and then in 50% formamide, 5 × SSC for 30 min. After washing in 2 × SSC, 0.2 × SSC, 0.2 × SSC each for 20 min, they were rinsed in 100 mM Tris-HCl, pH 7.5 and 150 mM NaCl. To reduce non-specific background, sections were incubated twice in 100 mM Tris-HCl, pH 7.5, 150 mM NaCl containing 0.2% Tween 20. The sections were incubated with alkaline phosphate-labelled anti-digoxigenin antibody at 1:300 dilution (Boehringer Mannheim) for 1 h at room temperature, and subsequently incubated with the enzyme reaction mixture (100 mM Tris-HCl, pH 9.5, 100 mM NaCl, 50 mM $MgCl_2$) containing nitroblue tetrazolium and 5-bromo-4-chloro-indolylphosphate (Boehringer Mannheim) several times at room temperature. The resultant sections were dehydrated, coverslipped and examined under a light microscope with bright-field illumination.

### Immunohistochemistry

Frozen tissue sections (4 μm thick) were air-dried, fixed with acetone at 4°C for 10 min and treated with PBS containing 0.03% hydrogen peroxide ($H_2O_2$) for 30 min at room temperature. After pre-incubation with PBS containing normal goat serum for 30 min at room temperature to block non-specific binding, sections were incubated with 1 μg $ml^{-1}$ affinity-purified rabbit polyclonal anti-TGF-$\beta_1$ antibody (Santa Cruz Biotechnology, Santa Cruz, CA, USA) in a humidified chamber at 4°C overnight. For a negative control, the anti-TGF-$\beta_1$ antibody was absorbed with excess immunized peptide (10 μg $ml^{-1}$) (Santa Cruz Biotechnology) for 2 h at room temperature. Next, tissue sections were washed thoroughly with PBS, incubated with biotinylated goat anti-rabbit IgG (Vector Laboratories, Burlingame, CA, USA) at room temperature for 40 min and then with biotin–avidin–peroxidase reagent (Vector Laboratories) at room temperature for 30 min. The bound immunocomplex was visualized by incubation with 0.02% 3,3′-deaminobenzidine tetrahydrochloride

A

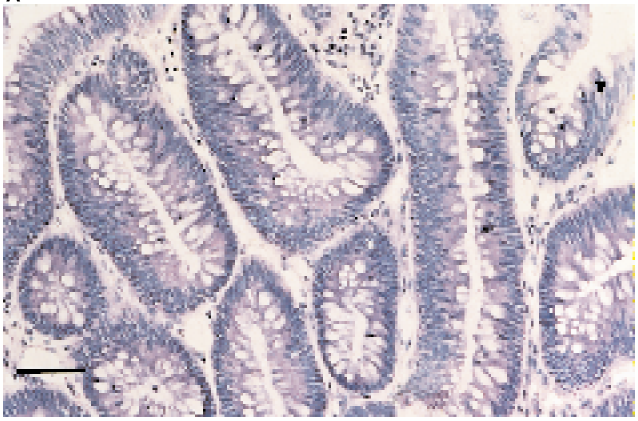

B

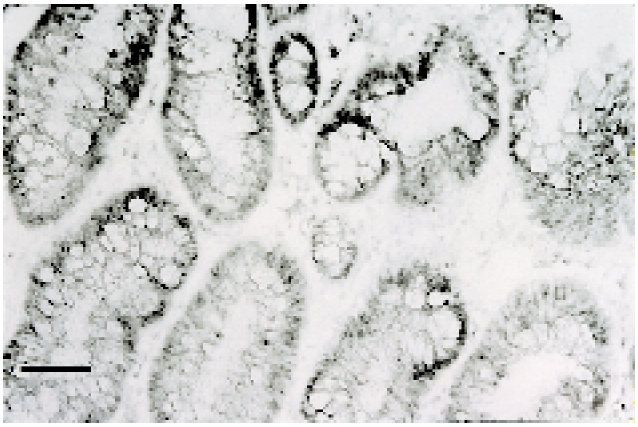

C

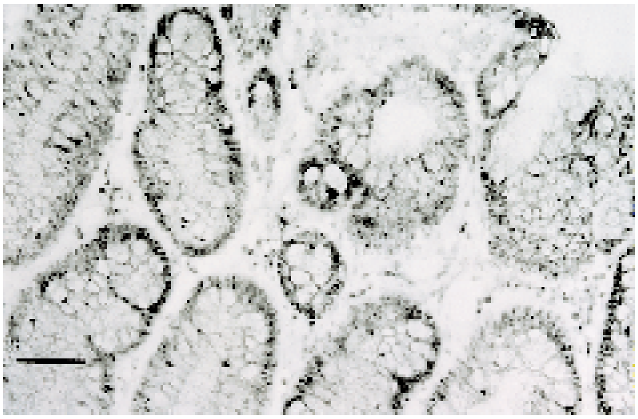

**Figure 2** Localization of TGF-β RI and TGF-β RII mRNAs in human colon adenoma by in situ hybridization. **(A)** Haematoxylin–eosin staining shows tubular adenoma with moderate atypia. **(B)** In situ hybridization with antisense probe to TGF-β RI shows high levels of mRNA prominently in human adenoma cells. **(C)** In situ hybridization with antisense probe to TGF-β RII shows a similar level of mRNA in the adenoma cells. Bars: 100 μm

© Cancer Research Campaign 1999

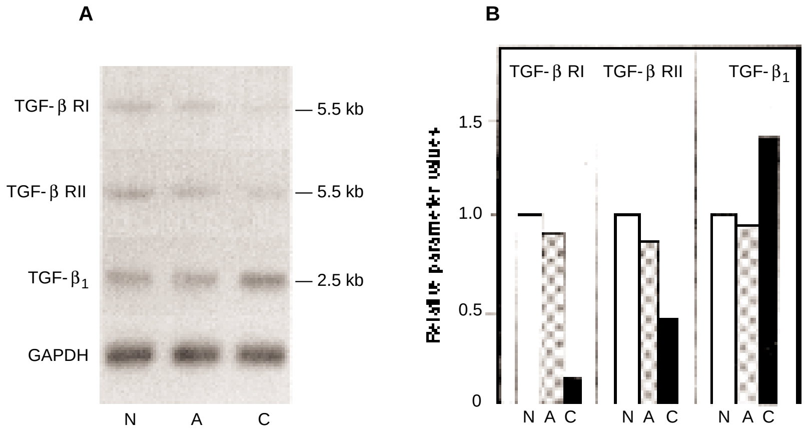

**Figure 3** Northern blot hybridization analyses of TGF-$\beta_1$, TGF-β RI and TGF-β RII mRNAs in normal colorectal mucosa, colorectal adenoma and colorectal cancer. **(A)** Northern blot hybridization was performed with poly-A RNAs (4 µg per lane) extracted from normal colorectal mucosa, colorectal adenoma and colorectal cancer. Blots were hybridized with $^{32}$P-labelled random-primed cDNA probes for GAPDH, TGF-$\beta_1$, TGF-β RI and TGF-β RII. The lower panel shows GAPDH mRNA expression. **(B)** The levels of TGF-$\beta_1$ and TGF-β receptor mRNAs in colorectal adenoma and colorectal cancer relative to normal colorectal mucosa were quantified by densitometric scanning. Normal colorectal mucosa was assigned a value of 1 and other values expressed relative to these were plotted. N, normal colorectal mucosa; A, colorectal adenoma; C, colorectal cancer

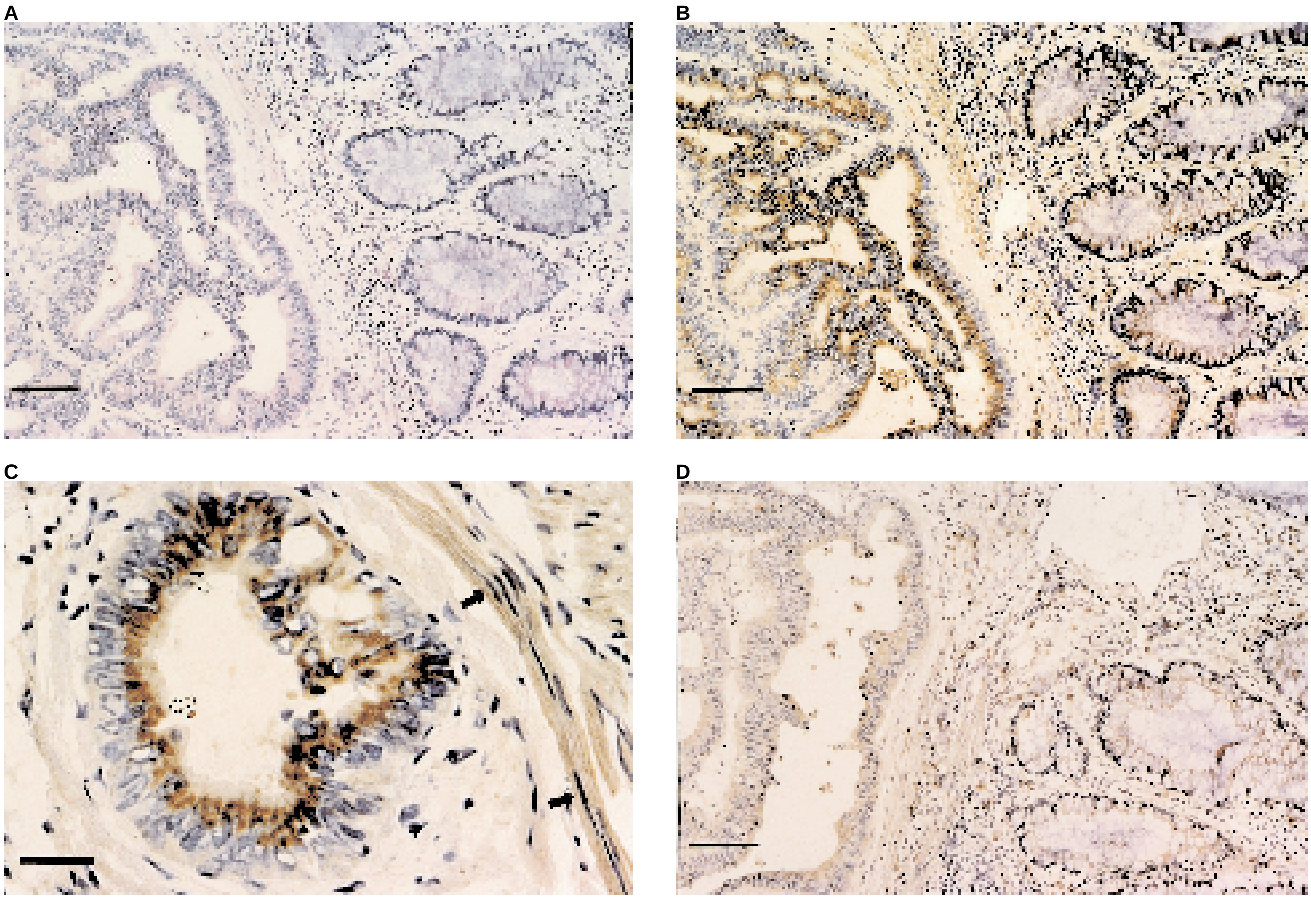

**Figure 4** Immunohistochemical staining with TGF-$\beta_1$ antibody in human colon cancer. **(A)** Haematoxylin–eosin staining of a sequential section shown in Figure 1, indicating cancer cells (left side), adjacent noninvolved normal colon mucosa (right side) and their interstitium. **(B)** The distribution of brown cytoplasmic staining of TGF-$\beta_1$ protein is shown in the human colon cancer cells and adjacent non-involved normal colon mucosa on a sequential section. There is weak immunostaining for TGF-$\beta_1$ in normal human colon crypts and interstitial cells. In contrast, strong immunostaining for TGF-$\beta_1$ is observed in the cytoplasm of human colon cancer cells. **(C)** High power views of a cancerous area on a sequential section show the strong signal for TGF-$\beta_1$ in the cytoplasm of human colon cancer (open arrow). Smooth muscle cells and the stroma in the connective tissue show fibrillar staining for TGF-$\beta_1$ (closed arrow). **(D)** The signal is specifically inhibited by the addition of excess TGF-$\beta_1$ peptide. Bars: **(A, B, D)** 100 µm; (**C**) 50 µm

 *© Cancer Research Campaign 1999*

in PBS containing 0.006% $H_2O_2$ for several minutes. Finally, the sections were counterstained lightly with Mayer haematoxylin (Sigma Diagnostics, St Louis, MO, USA).

### Interpretation of in situ hybridization and immunohistochemistry

Staining was scored independently by two observers (MM and KM), and a high level of concordance (90%) was achieved. In cases of disagreement, the slides were reviewed and a consensus view achieved. Staining was scored in a semiquantitative fashion from – to 2+: –, denoting no staining; ±, faint staining; +, moderate staining; 2+, strong staining (Table 1). For in situ hybridization of TGF-β receptors, we standardized the normal membranous pattern of staining as 2+. Because the staining pattern often varied within the same tumour, particularly when the degree of differentiation varied, the score was based on the dominant pattern.

### Northern blot hybridization

TGF-$\beta_1$, TGF-β RI and RII cDNAs used for in situ hybridization and 1.1 kb fragment of human glyceraldehyde-3-phosphate dehydrogenase (GAPDH; Clonetech, Palo Alto, CA, USA) were labelled with α-[$^{32}$P]-dCTP (3000 Ci mmol$^{-1}$; Amersham International) by the random primer labelling method. Total RNA was isolated from frozen tissues by extraction in guanidinium isothiocyanate–phenol–chloroform, and polyadenosine-A-rich RNA was selected using oligo (dT)-cellulose (New England Biolabs Inc., Beverly, MA, USA). Aliquots of 4 µg of poly-rich RNA were denatured with 2.2 M formaldehyde/50% formamide, electrophoresed through 1% agarose gels containing 2.2 M formaldehyde and transferred onto nylon membranes (Hybond-N+ RPN303B; Amersham International, Buckinghamshire, UK). The GAPDH cDNA was labelled with $^{32}$P (3000 Ci mmol$^{-1}$; Amersham International) by the random primer labelling method.

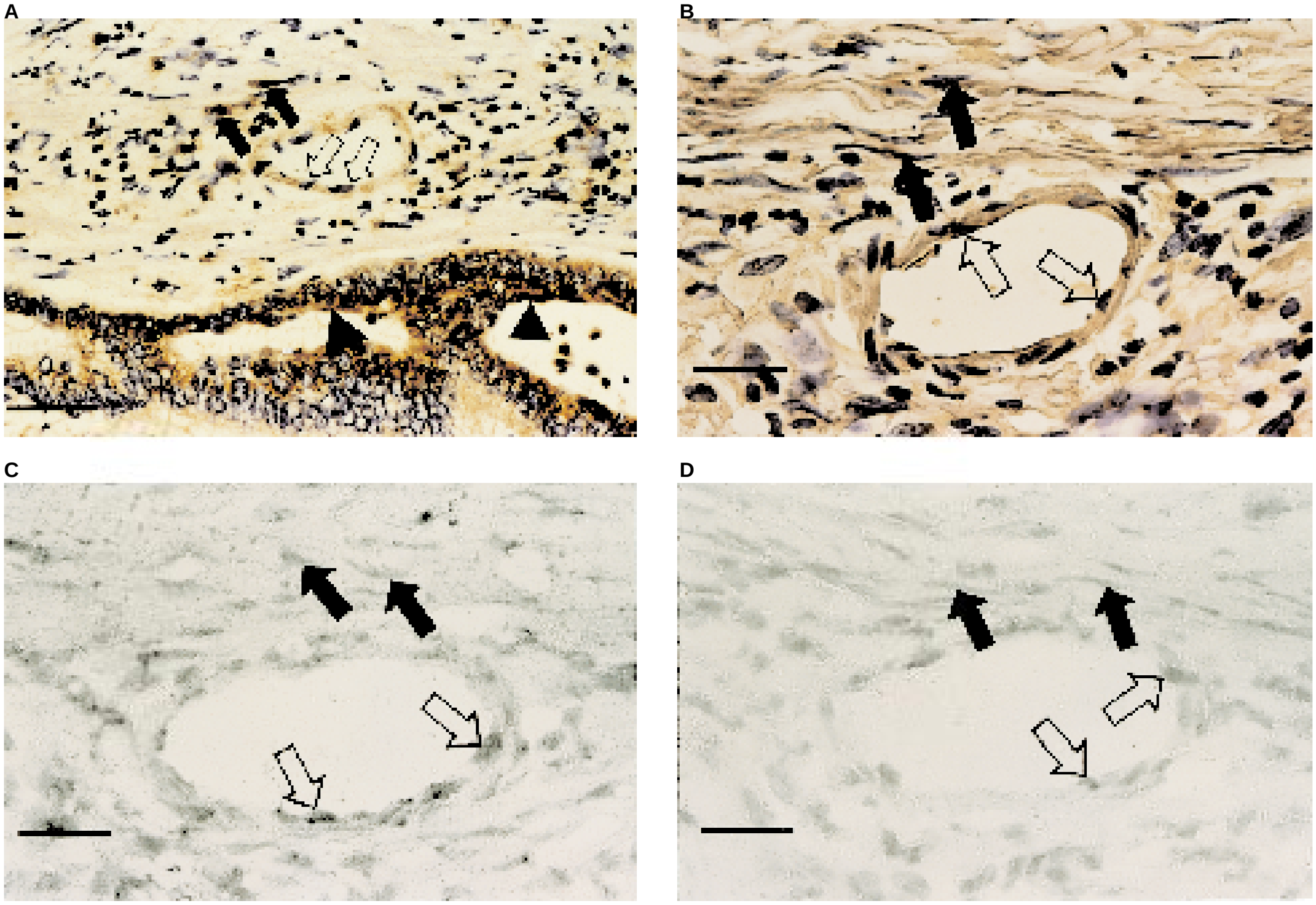

**Figure 5** Localization of TGF-β RI and TGF-β RII transcripts and TGF-$\beta_1$ protein in vimentin-positive fibroblast-like cells and endothelial cells in colorectal cancer stroma. **(A)** Immunohistochemistry with TGF-$\beta_1$ antibody shows that TGF-$\beta_1$ protein is localized in the colorectal cancer cells (arrowhead), the tumour-associated vascular endothelial cells (open arrow) and in the fibroblast-like cells (closed arrow) in the surrounding tissue stroma. Bars: 100 µm. **(B)** Immuno-histochemistry with vimentin antibody shows that the protein is also detected in the fibroblast-like cells (closed arrow) and endothelial cells (open arrow) in cancer stroma. Bar: 50 µm. (**C**) and **D**) Staining of the serial sections of cancer stroma with antisense riboprobe specific for TGF-β receptors. The grey stain indicates the location of TGF-β receptor messages. **(C)** Antisense section to show TGF-β RI message in endothelial cells (open arrow) adjacent to the colorectal cancer cells and the fibroblasts or myofibroblast cells (closed arrow) in colorectal cancer stroma, respectively. Cells expressing TGF-β RII mRNA were often closely associated with TGF-β RI expressing fibroblast cells and endothelial cells in colorectal cancer stroma (**D**). Bars: 50 µm

The filters were hybridized with the probe in solution containing 40% formamide, 5 × Denhardt's solution, 5 × saline–sodium phosphate–EDTA (SSPE), 0.5% sodium dodecyl sulphate (SDS) and 100 µg ml$^{-1}$ sonicated salmon testis DNA for 16 h at 42°C. After washing with 2 × SSC containing 0.1% SDS at 55°C, the filters were exposed to RX film (Fuji Photo Film Co., Kanagawa, Japan). After autoradiography, the filters were boiled in 0.5% SDS for 10 min to strip off the radioactive probes and rehybridized with $^{32}$P-labelled DNA probes for TGF-$\beta_1$, TGF-β RI and TGF-β RII in a similar manner.

### Immunofluorescence study

The affinity-purified rabbit polyclonal anti-Smad 4 antibody (Nakao et al, 1997) was a generous gift from Dr ten Dijke (Ludwig Institute for Cancer Research). After washing with PBS, the tumour specimens were minced with scissors and digested with Dulbecco's modified Eagle's medium (DMEM) containing 0.1% collagenase and dispase (Boehringer Mannheim, Mannheim, Germany) and incubated for 1 h at 37°C. Cells were then cultured onto LAB TEK chambers (Nunc, Naperville, IL, USA) and cultured for 1 week at 37°C in 5% carbon dioxide in air. Cells were washed with PBS and incubated for 1 h in DMEM in the presence or absence of 10 ng ml$^{-1}$ TGF-$\beta_1$ (R&D Systems, Minneapolis, MN, USA) at 37°C for 1 h. Slides were washed once with PBS, fixed with 4% para-formaldehyde/PBS containing 5% sucrose for 10 min, permeabilized with 0.2% Triton X100 in PBS for 30 s, and washed four times. To block non-specific binding, slides were incubated in PBS containing 10% normal goat serum for 30 min at room temperature and then with primary antibody at 1:1000 at 4°C for 16 h in a humidified chamber. After four washes, 1 µg ml$^{-1}$ FluoroLink™ Cy2™ labelled goat anti-rabbit IgG (H&L) (Amersham Life Science, Arlington Heights, IL, USA) was added, and the slides incubated for 30 min at room temperature with three subsequent washes. The slides were mounted with Perma Fluor™ Aqueous Mounting Medium (Shandon Lipshaw, Pittsburgh, PA, USA). The cells were observed by fluorescence microscope.

### RESULTS

### Colorectal cancer

#### *TGF-β receptor mRNA expression*

We summarized the histological classification, size, TNM classification and the grades of TGF-β RI, RII and TGF-$\beta_1$ transcripts and TGF-$\beta_1$ protein expressions in human colorectal cancer (Table 1).

Figure 1 shows differential expression patterns of TGF-β RI, RII and TGF-$\beta_1$ mRNA by in situ hybridization in human colon cancer and non-involved normal colon mucosa from patient no. 2 in Table 1. The strongest signal for TGF-β RI mRNA was found ubiquitously in all normal colorectal epithelial cells (Figure 1A). Cells expressing TGF-β RII mRNA were usually associated with TGF-β RI-expressing epithelial cells in normal colorectal mucosa (Figure 1C).

In contrast to the high level expression of TGF-β RI transcript in normal colorectal epithelial cells, colorectal cancer cells in 8/22 (38%) cases only faintly, if at all, expressed TGF-β RI mRNA (Table 1, Figure 1A). Moreover, faint TGF-β RII mRNA signals were observed in the cancer cells of 8/22 (38%) cases (Table 1, Figure 1C). The down-regulation was usually greater for TGF-β RI than for TGF-β RII, and 8/22 (38%) cases showed down-regulation of both receptor transcripts in the cancer cells (Table 1). We conclude that these signals are specific, because in situ hybridization with a sense probe to each TGF-β receptor showed no signal above background on a sequential section of the same sample (Figure 1B, D). It is notable that the distribution of mRNA expression for fibroblast growth factor receptor 1 (FGFR1), which promotes cell growth as an oncogene, was completely reversed compared with TGF-β receptor. Unlike the expression pattern of TGF-β receptor mRNAs, FGFR1 mRNA expression was not detected in normal colorectal epithelial cells, but progressively increased in cancer cells (data not shown). In other colorectal cancer cases, both TGF-β RI and RII mRNA signals were detected intensely in the cancer cells. The tumour size, pTNM classification and histological classification of colon cancer did not correlate with differences in the distribution of TGF-β receptor-expressing cells.

#### *TGF-$\beta_1$ mRNA expression*

The signal for TGF-$\beta_1$ mRNA was found ubiquitously in normal crypt. Strong and moderate signals of TGF-$\beta_1$ mRNA were observed in the cancer cells of 1/22 (5%) and 20/22 (90%) cases respectively (Table 1 and Figure 1E). In situ hybridization with a sense probe to TGF-$\beta_1$ showed no signal above background on a sequential section of the same sample (Figure 1F). The expression level of TGF-$\beta_1$ mRNA does not have any associations with those of TGF-β receptors (Table 1). TGF-$\beta_1$ mRNA was also occasionally expressed by inflammatory cells. TGF-$\beta_1$ mRNA was occasionally found in vascular endothelial and smooth muscle cells, but was absent in neutrophils (data not shown).

### Colorectal adenoma

Adenoma cells in 11/11 (100%) cases expressed TGF-β RI mRNA as strongly as normal colorectal epithelial cells did (Figure 2B). In addition, adenoma cells expressing TGF-β RII mRNA were often closely associated with TGF-β RI-expressing cells (Figure 2C). Strong signals for both TGF-β receptor mRNAs were found ubiquitously in colorectal adenoma cells, and there was no regional localization within the crypts. TGF-β receptors were expressed occasionally by inflammatory cells in the connective tissue. In addition, a moderate signal of TGF-$\beta_1$ mRNA was detectable in adenoma cells (data not shown). Moderate immunoreactivities with the TGF-$\beta_1$ antibody were observed in the adenoma cells (data not shown). Staining for TGF-$\beta_1$ was associated with all adenoma cells in cases with high expression levels of TGF-$\beta_1$, TGF-β RI and TGF-β RII mRNAs. In contrast to the faint expressions for TGF-β receptor transcripts in some colorectal cancer cells, the transcriptional and protein expression patterns of ligand and its receptors in adenoma cells were basically identical to those in normal colorectal cells. Moreover, these expression patterns did not vary with tumour differentiation.

### Northern blot hybridization analyses

To clarify the cell type-specific differences in expression of TGF-$\beta_1$ and TGF-β receptor mRNA between normal epithelial and colorectal tumours quantitatively, levels of TGF-$\beta_1$ and TGF-β receptor mRNAs from normal epithelial were compared to those for colorectal adenoma and cancer by Northern blot hybridization

 © Cancer Research Campaign 1999

analyses (Figure 3). We chose cancer cells from the patient in Figure 1, and adenoma cells from the patient in Figure 2. GAPDH expression was used as an internal control. The resulting differences in the expression of TGF-$\beta_1$, TGF-β RI and RII mRNAs shown in Figure 3A are graphically represented in Figure 3B.

A single 2.5 kb TGF-$\beta_1$ mRNA species was detected in poly-A mRNA isolated from normal epithelial, adenoma and cancer cells: poly-A fractions isolated from cancer cells showed more expression in normal epithelial and adenoma cell fractions. TGF-β RI mRNA (5.5 kb) and RII mRNA (5.5 kb) species were clearly detectable in normal epithelial and adenoma cells, but exhibited much lower expression levels in cancer cells (Figure 3A). Quantitation of the TGF-$\beta_1$, TGF-β RI and RII mRNAs by scanning densitometry (Figure 3B) also revealed that the levels of expression of TGF-β RI and RII mRNAs in cancer cells were 15% and 36% of normal epithelial cells, respectively, in spite of a slight increase of mRNA expression for TGF-$\beta_1$. In support of the in situ hybridization analyses shown in Figure 1, TGF-β receptor mRNA species were scarcely expressed in the colorectal cancer cells.

### *TGF-$\beta_1$ immunoreactivity*

Strong and moderate immunoreactivities with the anti-TGF-$\beta_1$ antibody were observed in the colorectal cancer cells of all cases. (Table 1, Figure 4B and 4C). TGF-$\beta_1$ in the cancer cells was stained more intensely than that in normal epithelial cells. The antibody absorbed with excess TGF-$\beta_1$ peptide does not react with the antigen on the tissue sections (Figure 4D). Immunohistochemical staining for TGF-$\beta_1$ protein was associated with all cancer cells in cases with high expression levels of TGF-$\beta_1$ mRNA and the expression of TGF-β RII mRNA even without expression of TGF-β RI transcript (Table 1, Figure 1 and 4). In addition, lamina propria and the collagen fibre in the connective tissue occasionally showed fibrillar staining for TGF-β (Figure 4C).

### Expressions of TGF-β receptor mRNAs and the deposition of TGF-$\beta_1$ protein in vimentin-positive fibroblast-like cells and endothelial cells in the colorectal cancer stroma

Next, we focused on cancer and stromal interactions, and studied the expressions of TGF-β receptor transcripts as well as the location of TGF-$\beta_1$ protein in the colorectal cancer stroma.

Figure 5A illustrates the staining of the colorectal cancer tissue for TGF-$\beta_1$ protein and indicates that TGF-$\beta_1$ protein was deposited in the malignant carcinoma cells (arrowhead), the tumour-associated vascular endothelial cells (open arrow) and in the fibroblast-like cells (closed arrow) in the surrounding tissue stroma. In addition, the results of in situ hybridization revealed a

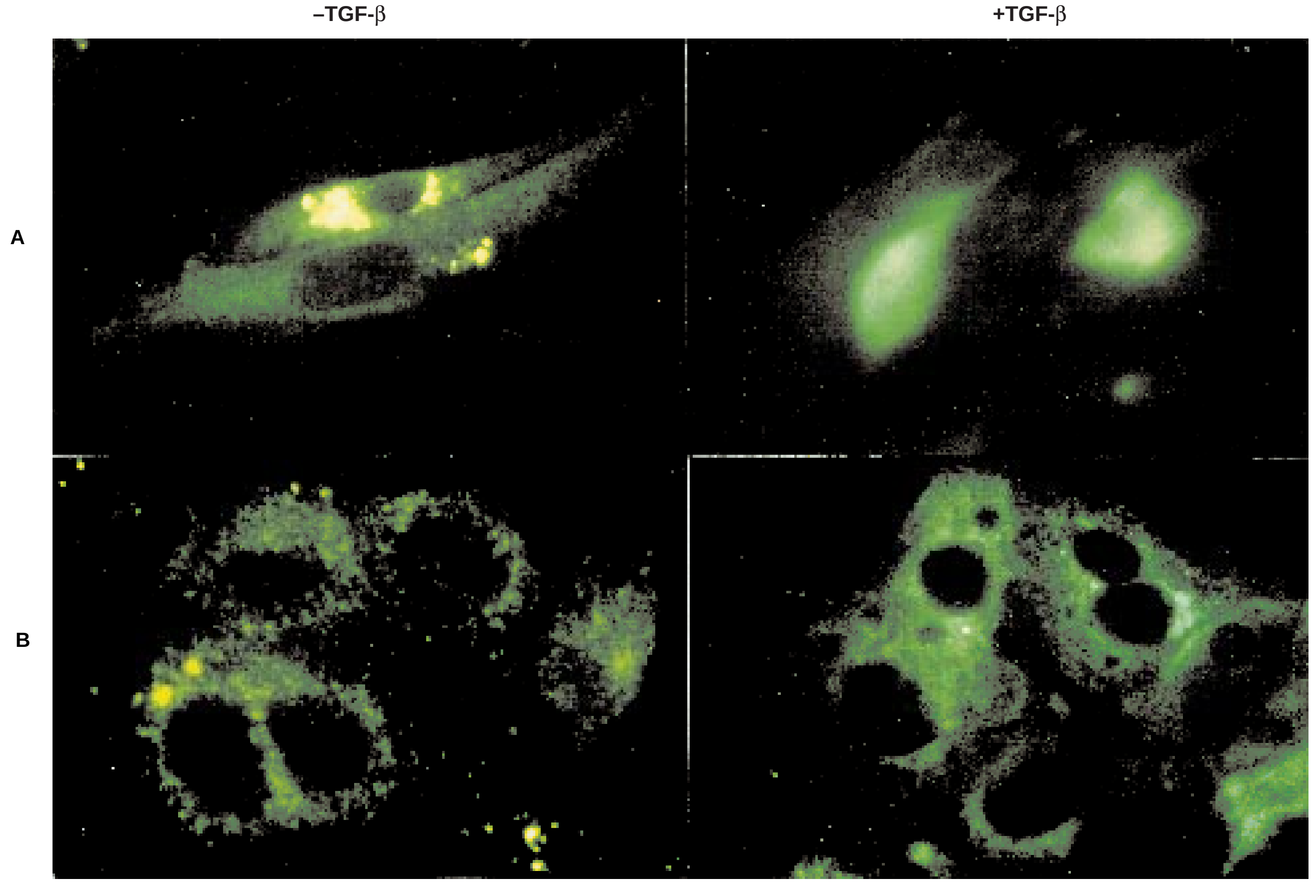

**Figure 6** Immunofluorescence study of Smad4 in colorectal adenoma and cancer cells after TGF-β stimulation. Colorectal adenoma and cancer cells were incubated in the absence or presence of TGF-β for 1 h. Smad 4 was localized in the cells by immunofluorescence using specific antisera. Smad 4 staining was predominant in the cytoplasm in the absence of TGF-β. After TGF-β stimulation, nuclear staining for Smad 4 was observed in adenoma cells (**A**), by contrast, the staining remained predominant in the cytoplasm of cancer cells (**B**)

moderate reaction with the riboprobe for TGF-β RI, which suggests that vimentin-positive vascular endothelial cells (open arrow) and irregular, spindle-shaped, fibroblast-like cells (closed arrow) were synthesizing messages for TGF-β RI (Figure 5B, C). Cells expressing TGF-β RII mRNA were often closely associated with TGF-β RI-expressing fibroblast-like cells and endothelial cells in colorectal cancer stroma (Figure 5D). The observation that endothelial cells and fibroblast-like cells in the cancer stroma synthesize TGF-β receptors, coupled with immunohistochemical staining of TGF-$\beta_1$ in these cells and the strong or moderate expression of TGF-$\beta_1$ transcripts in the colorectal cancer cells (Table 1, Figure 1E and Figure 3), suggests that the endothelial cells and the fibloblast-like cells have potential ability to bind TGF-β secreted from the colorectal cancer cells.

### Translocation of Smad 4 in colorectal adenoma cell and cancer cells after stimulation with TGF-$\beta_1$

Signalling by TGF-β receptors is mediated by the recently identified Smad protein family. The activated TGF-β receptor induces phosphorylation of two such proteins, Smad 2 and Smad 3, which form hetero-oligomeric complexes with Smad 4 that translocate to the nucleus, where they then regulate transcriptional responses.

To study for TGF-β signal transduction in adenoma cells and colorectal cancer cells directly, we first cultured adenoma cells, which have both TGF-β receptors, and colorectal cancer cells from patient no. 4, who expressed TGF-β RI faintly. Northern blot analyses with Smad-specific probes revealed the Smad 2, 3 and 4 mRNA expressions, and no mutations of these Smads were observed (data not shown). Then, we investigated whether treatment of these cells with TGF-β leads to an accumulation of Smad 4 protein in the nucleus.

The subcellular localization of Smad 4 in colorectal adenoma cells and cancer cells before or after stimulation with TGF-β was analysed by immunofluorescence using specific antisera. As a control, rabbit IgG did not react with the antigen on the cells (data not shown). In the absence of ligand, staining for Smad 4 was seen predominantly in the cytoplasm of the cells, whereas after stimulation with TGF-$\beta_1$ for 1 h, the staining in adenoma significantly accumulated in the nucleus (Figure 6A). In contrast, the staining remained unaltered in the cytoplasm of the cancer cells upon TGF-$\beta_1$ stimulation (Figure 6B). These results indicated that the colorectal cancer cells expressing only faintly TGF-β RI, cannot transduce TGF-β signal, but the adenoma cells can.

### DISCUSSION

### TGF-β in normal colorectal epithelial cell

Our results demonstrated that both TGF-β RI and RII mRNAs were strongly expressed in normal colorectal epithelial cell. In situ hybridization and immunohistochemistry of the ligand also suggested that TGF-$\beta_1$ is produced by, and is present within, the colorectal epithelium, which is consistent with previous reports (Barnard et al, 1989; Koyama et al, 1989; Avery et al, 1993; Barnard et al, 1993). TGF-β inhibits the growth of rat intestinal (jejunal) epithelial cell line (Kurokawa et al, 1987; Barnard et al, 1989) and can directly induce apoptoic cell death (Cui et al, 1995). Thus, TGF-β may play an important role in mediating differentiation and cell death in an autocrine fashion.

### TGF-β in colorectal adenoma

As in the normal crypts, there was codistribution of TGF-β RI and TGF-β RII within epithelial cells, with similar staining intensity for both of these receptors. Furthermore, Smad 4 translocated into nucleus on treatment of the adenoma cells with TGF-β. Taken together with the recent finding that TGF-β is a suppresser of colorectal adenoma cells in vitro (Manning et al, 1991), our results lead to the conclusion that the signal transduction pathway of TGF-β is intact in colorectal adenoma cells.

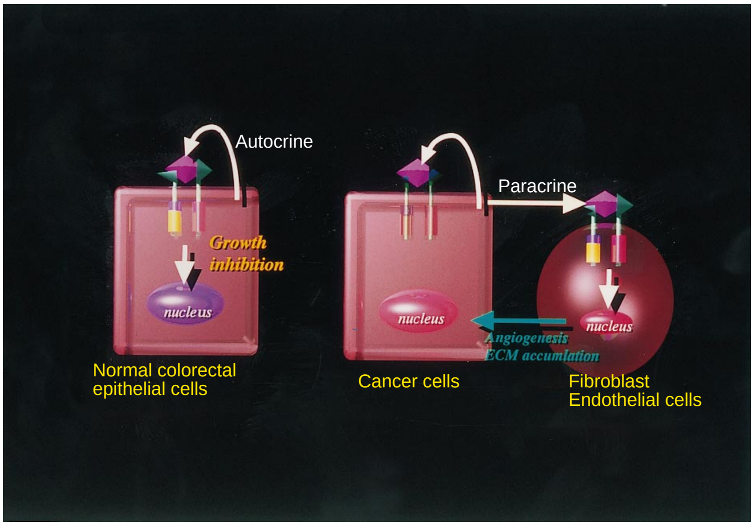

**Figure 7** Differential sensitivity of TGF-β for normal colorectal epithelial/adenoma cells and cancer cells. In normal colorectal epithelial/adenoma cells, TGF-β inhibits cell growth through TGF-β receptors by an autocrine mechanism. Without TGF-β receptors in cancer cells, its growth-inhibitory signal cannot be propagated. TGF-β secreted from colorectal cancer cells is involved in angiogenesis and stroma formation by binding to TGF-β receptors on endothelial cells and fibroblast

 © Cancer Research Campaign 1999

However, using APC knockout mouse, Oshima et al (1997) and Zhang et al (1997) reported the distribution of TGF-β RII in normal gut and its down-regulation in intestinal adenoma. In addition, Akiyama et al (1997) found that eight of 13 replication error phenotype (RER)-positive adenomas had TGF-β RII mutations in the polyadenine tract and these mutations were present in all adenomas with moderate atypia. These reports suggest that TGF-β RII inactivation is an early event contributing to the formation of premalignant colon neoplasms. Although we sequenced TGF-β RII cDNA from ten adenoma tissues, no mutants were found (data not shown).

### TGF-β and malignant progression

Many transformed cell lines compared with their normal counterparts are resistant to the growth-inhibitory effects of TGF-β. However, it remains to be elucidated which mechanism of TGF-β resistance occurs in human tumour cells as well as at which step in the multistep process of carcinogenesis cells become resistant to TGF-β.

To understand the functional implications of these mechanisms in vivo, we performed in situ hybridization and Northern blot hybridization for TGF-β receptors. This work for the first time extends studies on the transcriptional levels of TGF-β receptors in human tissues to colorectal cancer, and shows that TGF-β receptor mRNAs were only faintly expressed in eight of 22 colorectal cancers, and that down-regulation was greater for TGF-β RI than for TGF-β RII (Figure 1A, C and Figure 3). As for the meaning of down-regulation of TGF-β RI, Wang et al (1996) reported that low TGF-β RI expression levels in a human colon carcinoma cell can be a limiting factor for TGF-β response and autocrine-negative activity, which was supported by evidence that increased TGF-β RI expression in the cell line with a low level of TGF-β RI expression increased TGF-β responsiveness as well as that autocrine-negative activity reduced in vivo malignancy. These are the major mechanisms for TGF-β resistance in human colorectal cancer.

Our findings demonstrate that reduced TGF-β receptor transcripts develop relatively late in the evolution of colorectal cancer. Furthermore, treatment of colorectal cancer cells with TGF-β does not lead to an accumulation of Smad 4 protein in the nucleus, although the translocation was seen in adenoma cells. These results confirm and extend previous findings that the conversion of the non-tumorigenic phenotype of human colonic adenoma cell line to a tumorigenic phenotype is accompanied by a reduced response to the growth inhibitory effects of TGF-β (Manning et al, 1991); moreover, intestinal polyps developed into more malignant tumour than those in the single $Apc^{\Delta 716}$ heterozygote by induction of Smad 4 mutation into the $Apc^{\Delta 716}$ knockout mouse (Takaku et al, 1998). Therefore, abnormalities of the TGF-β signal transduction pathway, including receptors and Smad, play a significant role in the malignant progression of colorectal cancer.

Immunohistochemical staining for TGF-$\beta_1$ protein was associated with all cancer cells in cases with high expression levels of TGF-$\beta_1$ mRNA and the expression of TGF-β RII mRNA even without expression of TGF-β RI transcript. This can be explained by previous reports and our own findings that the homodimer of TGF-β RII binds to its ligand in the absence of TGF-β RI, however, TGF-β can only induce its multiple biological actions through the heterodimic complex of TGF-β RI and TGF-β RII (Attisano et al, 1994).

Although the mechanism of reduction of TGF-β RI transcripts is currently unknown, several reports analysed the promoter region of TGF-β RII to pursue the transcriptional mechanism of this gene. Bae et al (1995) identified two positive regulatory elements (PRE1 and PRE2) and at least one negative regulatory element (NRE) in the promoter region of TGF-β RII. Furthermore, Kim et al (1997) reported that an absolute reduction of TGF-β RII mRNA followed by transformation of keratinocytes with E1A oncogene is the result of decreased expression of unidentified transcription factor complexes that interact with PRE1 and PRE2. Therefore, we speculate that the down-regulation of TGF-β RII in human colorectal cancer is caused by a decrease of these transcription factors. Furthermore, our findings, as presented in the current study, demonstrate that a transient down-regulation of TGF-β RII occurs in hepatocytes; however, the levels of TGF-β RII expression remain high in non-parenchymal cells during liver regeneration (Date et al, 1998). These results lead us to conclude that these transcription factors are specific to epithelial cells.

Our results demonstrate that human colorectal cancer cells express higher levels of TGF-$\beta_1$ transcripts than their normal counterparts. This raises the question of which biological functions of TGF-β secreted from human colorectal cancer have a positive effect on cancer development if the cancer cells lose the ability to respond to the peptide. It has been postulated that TGF-β stimulates tumour growth indirectly via paracrine effects on stromal elements of the tumour. Our results support this hypothesis because the expression of TGF-β receptors was observed in mesenchymal cells such as fibroblasts and endothelial cells. TGF-β induces the net accumulation of extracellular matrix protein not only by elevating extracellular matrix protein expression but also by strongly up-regulating the inhibitors of extracellular matrix degrading enzymes and down-regulating extracellular matrix degrading enzymes (Ignotz et al, 1986; Laiho et al, 1986; Edwards et al, 1987). Directly, or through other cells that it attracts and stimulates, TGF-$\beta_1$ can also induce formation of new blood vessels in vivo (Roberts et al, 1986). This response might seem paradoxical given the strong growth inhibitory effect of TGF-$\beta_1$ and TGF-$\beta_3$ on endothelial cell monolayer cultures (Fráter-Schröder et al, 1986; Heinmark et al, 1986). However, it should be noted that, under certain culture conditions, TGF-β does not inhibit endothelial cell growth, and endothelial cells tend to organize into tubular structures reminiscent of an angiogenic process (Madri et al, 1988).

In summary, down-regulation of TGF-β receptors may play a corresponding role in development of the resistance to the growth-inhibitory effects of TGF-β, that is, aggressive cell growth, demonstrated by human colorectal cancer. Moreover, the TGF-$\beta_1$ production in colorectal cancer as well as the presence of TGF-β receptors in endothelial cells and fibroblasts around colorectal cancer cells strongly suggests that this multipotential cytokine is involved in angiogenesis and stroma formation (Figure 7).

### ACKNOWLEDGMENTS

We thank Dr SW Qian (National Cancer Institute), Dr K Miyazono (Ludwig Institute for Cancer Research), Dr HF Lodish (Whitehead Institute for Biomedical Research) and Dr P ten Dijke for generously donating TGF-$\beta_1$ cDNA, TGF-β RI cDNA, TGF-β RII cDNA and anti-Smad 4 antibody, respectively, and surgeons of the Second Department of Surgery (Kansai Medical University) for cooperation in this study. This study was supported by a grant-in aid for scientific research from the Ministry of Education, Science and Culture of Japan.

© Cancer Research Campaign 1999

### REFERENCES

Attisano L, Wrana JL, López-Casillas F and Massagué J (1994) TGF-β receptors and actions. *BBA* **1222**: 71–80

Avery A, Paraskeva C, Hall P, Flanders KC, Sporn M and Moorghen M (1993) TGF-β expression in the human colon: differential immunostaining along crypt epithelium. *Br J Cancer* **68**: 137–139

Bae HW, Geiser AG, Kim DH, Chung MT, Burmester JK, Sporn MB, Roberts AB and Kim SJ (1995) Characterization of the promoter region of the human transforming growth factor-β type II receptor gene. *J Biol Chem* **270**: 29460–29468

Barnard JA, Beauchamp RD, Coffey RJ and Moses HL (1989) Regulation of intestinal epithelial cell growth by transforming growth factor type β. *Proc Natl Acad Sci USA* **86**: 1578–1582

Barnard JA, Lyons RM and Moses HL (1990) The cell biology of transforming growth factor β. *Biochem Biophys Acta* **1032**: 79–87

Barnard JA, Warwick GJ and Gold LI (1993) Localization of transforming growth factor β isoforms in the normal murine small intestine and colon. *Gastroenterology* **105**: 67–73

Birchenall-Roberts MC, Ruscetti FW, Kasper J, Lee HD, Friedman R, Geiser A, Sporn MB, Roberts AB and Kim SJ (1995) Transcriptional regulation of the transforming growth factor $\beta_1$ promoter by *v-src* gene products is mediated through the AP-1 complex. *Mol Cell Biol* **10**: 4978–4983

Cui W, Fowlis DJ, Cousins FM, Duffie E, Bryson S, Balmain A and Akhurst RJ (1995) Concerted action of TGF-$\beta_1$ and its type II receptor in control of epidermal homeostasis in transgenic mice. *Genes Dev* **9**: 945–955

Date M, Matsuzaki K, Matsushita M, Sakitani K, Shibano K, Okajima A, Yamamoto C, Ogata N, Okumura T, Seki T, Kubota Y, Kan M, McKeehan WL and Inoue K (1998) Differential expression of transforming growth factor-β and its receptors in hepatocytes and nonparenchymal cells of rat liver after CC14 administration. *J Hepatol* **28**: 572–581

Edwards DR, Murphy G, Reynolds JJ, Whitham SE, Docherty AJP, Angel P and Heath JK (1987) Transforming growth factor beta modulates the expression of collagenase and metalloproteinase inhibitor. *EMBO J* **6**: 1899–1904

Fráter-Schröder M, Müller G, Birchmeier W and Böhlen P (1986) Transforming growth factor-beta inhibits endothelial cell proliferation. *Biochem Biophys Res Commun* **137**: 295–302

Franzen P, ten Dijke P, Yamashita H, Schulz P, Heldin C-H and Miyazono K (1993) Cloning of a TGF-β type I receptor that forms a heteromeric complex with the TGF-β type II receptor. *Cell* **75**: 681–692

He WW, Gustafson ML, Hirose S and Donahoe PK (1993) Developmental expression of four novel serine/threonine kinase receptors homologous to the activin/transforming growth factor-β type II receptor family. *Den Dyn* **196**: 133–142

Heinmark RL, Twardzik DR and Schwartz SM (1986) Inhibition of endothelial regeneration by type-beta transforming growth factor from platelets. *Science* **233**: 1078–1080

Hoosein NM, McKnight MK, Levine AE, Mulder KM, Childress KE, Brattain DE and Brattain MG (1989) Differential sensitivity of subclass of human colon carcinoma cell lines to the growth inhibitory effects of transforming growth factor-$\beta_1$. *Exp Cell Res* **181**: 442–453

Hutter RVP and Sobin LH (1986) A universal staging system for cancer of the colon and rectum. *Arch Pathol Lab Med* **110**: 367–368

Ignotz RA and Massagué J (1986) Transforming growth factor-β stimulates the expression of fibronectin and collagen and their incorporation into the extracellular matrix. *J Biol Chem* **261**: 4337–4345

Kim DH, Chang JH, Lee KH, Lee HY and Kim SJ (1997) Mechanism of E1A-induced transforming growth factor-β (TGF-β) resistance in mouse keratinocytes involves repression of TGF-β type II transcription. *J Biol Chem* **272**: 688–694

Koyama S and Podolsky DK (1989) Differential expression of transforming growth factors α and β in rat intestinal epithelial cells. *J Clin Invest* **83**: 1768–1773

Kurokawa M, Lynch K and Podolsky DK (1987) Effects of growth factors on an intestinal epithelial cell line: transforming growth factor β inhibits proliferation and stimulates differentiation. *Biochem Biophys Res Commun* **142**: 775–782

Laiho M, Saksela O, Andreasen PA and Keski-Oja J (1986) Enhanced production and extracellular deposition of the endothelial-type plasminogen activator inhibitor in cultured human lung fibroblast by transforming growth factor-β. *J Cell Biol* **103**: 2403–2410

Lin HY, Wang X-F, Ng-Eaton E, Weinberg RA and Lodish HF (1992) Expression cloning of the TGF-β type II receptor, a functional transmembrane serine/threonine kinase. *Cell* **68**: 775–785

Madri JA, Pratt BM and Tucker AM (1988) Phenotypic modulation of endothelial cells by transforming growth factor-β depends upon the composition and organization of the extracellular matrix. *J Cell Biol* **106**: 1375–1384

Manning AM, Williams AC, Game SM and Paraskeva C (1991) Differential sensitivity of human colonic adenoma and carcinoma cells to transforming growth factor β (TGF-β): conversion of an adenoma cell line to a tumorigenic phenotype is accompanied by a reduced response to the inhibitory effects of TGF-β. *Oncogene* **6**: 1471–1476

Markowitz S, Wang J, Myeroff L, Parsons R, Sun L, Lutterbaugh J, Fan RS, Zborowska E, Kinzler KW, Vogelstein B, Brattain M and Willson JKV (1995) Inactivation of the type II TGF-β receptor in colon cancer cells with microsatellite instability. *Science* **268**: 1336–1338

Massagué J, Hata A and Liu F (1997) TGF-β signalling through the Smad pathway. *Trends Cell Biol* **7**: 187–192

Mathew LS and Vale WW (1991) Expression cloning of an activin receptor, a predicted transmembrane serine kinase. *Cell* **65**: 973–982

Matsuzaki K, Xu J, Wang F, McKeehan WL, Krummen L and Kan M (1993) A widely expressed transmembrane serine/threonine kinase that does not bind activin, inhibin, transforming growth factor β, or bone morphogenic factor. *J Biol Chem* **268**: 12719–12723

Matsuzaki K, Kan M and McKeeham WL (1996) Ligand-dependent and independent interactions in assembly of a pentameric transforming growth factor beta receptor complex. *In Vitro Cell Dev Biol* **32**: 345–360

Muto T, Bussey HJR and Morson BC (1975) The evolution of cancer of the colon and rectum. *Cancer* **36**: 2251–2270

Nakao A, Imamura T, Souchelnytskyi S, Kawabata M, Isisaki A, Oeda E, Tamaki K, Hanai J, Heldin CH, Miyazono K and ten Dijke P (1997) TGF-β receptor-mediated signaling through Smad2, Smad3 and Smad4. *EMBO J* **16**: 5353–5362

Oshima H, Oshima M, Kobayashi M, Tsutsumi M and Makoto MT (1997) Morphological and molecular processes of polyp formation in $Apc^{\Delta 716}$ knockout mice. *Cancer Res* **57**: 1644–1649

Qian SW, Kondaiah P, Roberts AB and Sporn MB (1990) cDNA cloning PCR of rat transforming growth factor $\beta_1$. *Nucleic Acids Res* **18**: 3059

Roberts AB, Anzano MA, Wakefield LM, Roche NS, Stern DF and Sporn MB (1985) Type β: transforming growth factor: a bifunctional regulator of cellular growth. *Proc Natl Acad Sci USA* **82**: 119–123

Roberts AB, Sporn MB, Assoian RK, Smith JM, Roche NS, Wakefield LM, Heine UI, Liotta LA, Falanga V, Kehrl JH and Fauci AS (1986) Transforming growth factor type β rapid induction of fibrosis and angiogenesis in vivo and stimulation of collagen formation in vitro. *Proc Natl Acad Sci USA* **83**: 4167–4171

Serra R and Moses HL (1996) Tumor suppressor genes in the TGF-β signaling pathway? *Nature Med* **2**: 390–391

Sporn MB and Roberts AB (1988) Peptide growth factors are multifunctional. *Nature* **332**: 217–219

Sporn MB and Roberts AB (1992) Transforming growth factor-β: recent progress and new challenges. *J Cell Biol* **119**: 1017–1021

Takaku K, Oshima M, Miyoshi H, Matsui M, Michael FS and Makoto MT (1998) Intestinal tumorigenesis in compound mutant mice of both Dpc4 (Smad4) and Apc genes. *Cell* 92: 645–656

ten Dijke P, Ichijo H, Franzén P, Schulz P, Saras J, Toyoshima H, Heldin C-H and Miyazono K (1993) Activin receptor-like kinases: a novel subclass of cell-surface receptors with preedicted serine/threonine kinase activity. *Oncogene* **8**: 2879–2887

ten Dijke P, Yamashita H, Ichijo H, Franzén P, Laiho M, Miyazono K and Heldin C-H (1994) Characterization of type I receptors for transforming growth factor-β and activin. *Science* **204**: 101–104

Tsushima H, Kawata S, Tamura S, Ito N, Shirai Y, Kiso S, Imai Y, Shimomukai H, Nomura Y, Matsuda Y and Matsuzawa Y (1996) High levels of transforming growth factor $\beta_1$ in patients with colorectal cancer: association with disease progression. *Gastroenterology* **110**: 375–382

Wang J, Han W, Zborowska E, Liang J, Wang X, Willson JKV, Sun L and Brattain MG (1996) Reduced expression of transforming growth factor β type I receptor contributes to the malignancy of human colon carcinoma cells. *J Biol Chem* **271**: 17366–17371

Xu J, Matsuzaki K, McKeehan K, Wang F, Kan M and McKeehan WL (1994) Genomic structure and cloned cDNAs predict that four variants in the kinase domain of serine/threonine kinase receptors arise by alternative splicing and poly (A) addition. *Proc Natl Acad Sci USA* **91**: 7957–7961

*© Cancer Research Campaign 1999*

Xu J, McKeehan K, Matsuzaki K and McKeehan WL (1995) Inhibin antagonizes inhibition of liver cell growth by activin by a dominant-negative mechanism. *J Biol Chem* **270**: 6308–6313
Zhang T, Nanney LB, Peeler MO, Williams CS, Lamps L, Heppner KJ, DuBois RN and Beauchamp D (1997) Decreased transforming growth factor β type II receptor expression in intenstinal adenomas from *Min*/+ mice is associated with increased cyclin D1 and cyclin-dependent kinase 4 expression. *Cancer Res* **57**: 1638–1643

© Cancer Research Campaign 1999
